# The genus *Madurella*: Molecular identification and epidemiology in Sudan

**DOI:** 10.1371/journal.pntd.0008420

**Published:** 2020-07-30

**Authors:** Elhadi A. Ahmed, Bakri Y. M. Nour, Adam D. Abakar, Samirah Hamid, Ahmed A. Mohamadani, Mohamed Daffalla, Mogahid Mahmoud, Hisham N. Altayb, Marie Desnos-Ollivier, Sybren de Hoog, Sarah A. Ahmed

**Affiliations:** 1 Department of Medical Microbiology, Faculty of Medical Laboratory Sciences, University of Gezira, Sudan; 2 Blue Nile National Institute for Communicable Diseases (BNNICD), University of Gezira, Sudan; 3 Department of Medical Parasitology, Faculty of Medical Laboratory Sciences, University of Gezira, Sudan; 4 Department of Pathology, Faculty of Medicine, University of Gezira, Sudan; 5 Department of Surgery, Faculty of Medicine, University of Gezira, Sudan; 6 Biochemistry Department, Faculty of Sciences, King Abdulaziz University, Saudi Arabia; 7 Institut Pasteur, Molecular Mycology Unit, National Reference Center for Invasive Mycoses and Antifungals, Paris, France; 8 Foundation Atlas of Clinical Fungi, Hilversum, The Netherlands; 9 Center of Expertise in Mycology of Radboud University Medical Center / Canisius Wilhelmina Hospital, Nijmegen, The Netherlands; 10 Faculty of Medical Laboratory Sciences, University of Khartoum, Khartoum, Sudan; Faculty of Science, Ain Shams University (ASU), EGYPT

## Abstract

Eumycetoma (mycotic mycetoma) is the fungal form of mycetoma, a subcutaneous infection occurring in individuals living in endemic areas of the disease. The Sudan is hyperendemic for mycetoma, with the highest incidence being reported from Gezira State, Central Sudan. The present study was conducted at the Gezira Mycetoma Center and aimed to determine the cause of black-grain eumycetoma in the state and describe its epidemiology. Black-grain specimens were collected during the surgical operation and direct detection of the causative agent was performed using *M*. *mycetomatis* species-specific PCR and ITS PCR followed by sequencing. Black-grain was reported from 93.3% of all confirmed mycetoma cases (n = 111/119), with a prevalence in young males. Of the 91 samples subjected to direct PCR, 90.1% (n = 82) gave positive results. The predominant species (88.2%) was *Madurella mycetomatis*. One sample was identified as *M*. *fahalii*, one as *M*. *tropicana*, and one matched the phytopathogenic species *Sphaerulina rhododendricola*. The highest endemic zones were Southern Gezira (76.6%) and Northern Sinnar (23.4%). The study confirmed that direct molecular detection on grains provides rapid and specific diagnosis of agents of eumycetoma.

## Author summary

Eumycetoma is a neglected fungal disease endemic in Africa, India, and Latin America. Black-grain eumycetoma is the most common type in Africa and is mainly caused by *Madurella* spp. The Sudan, and in particular Gezira State, central Sudan is hyperendemic for black-grain eumycetoma. Patients with this type of mycetoma are treated with surgery in combination with antifungal therapy. In this study, we collected surgical biopsies from patients attending Gezira Mycetoma Center to directly identify the etiology of black-grain eumycetoma in this state. We also studied the epidemiology of the disease based on the demography of the patients’ population. Our result showed that the highest endemic regions were Southern Gezira (76.6%) and Northern Sinnar (23.4%). By applying direct PCR and sequencing we confirmed that the most common etiology of the disease is *Madurella mycetomatis* (88.2%). In addition, we found one case of *M*. *fahalii* and the first Sudanese case of *M*. *tropicana* and *Sphaerulina rhododendricola*.

## Introduction

Black-grain mycetoma is caused exclusively by fungi, and to date around 23 species have been reported as being responsible for this infection [1, 2, 3]. Agents of black grain mycetoma are affiliated to three orders of Ascomycota, *viz*. *Pleosporales*, *Sordariales* and *Chaetothyriales* [1]. Although eight species of *Chaetothyriales*, belonging to *Exophiala* and *Cladophialophora* have been incriminated in mycetoma, members of this order are less frequent. The largest diversity of black-grain agents is found in *Pleosporales*: ten species belonging to eight genera have been reported [1,4, 5, 6]. In contrast, *Sordariales* harbor only a small number of species able to cause mycetoma, but among these are the predominant agents worldwide in the genus *Madurella* [7, 8].

Irrespective of phylogenetic positions, all agents of black-grain mycetoma produce similar clinical features, *i*.*e*. progressive tumefaction, sinuses, and black grains in discharge or embedded in tissue [9]. For this reason, it is difficult to differentiate the 23 species histologically [1,10]. After culturing of the grains, members of *Chaetothyriales* produce dark olivaceous black colonies and microscopically yeast cells or more or less characteristic conidia [4]. Species of *Pleosporales* produce dark grey or olive green velvety or woolly colonies, but clinical strains may remain sterile or produce ascomata or conidiomata with delay [6, 11]. Members of *Sordariales* produce brownish to dark yellow or grey colonies which are mostly cerebriform and glabrous or with very short aerial mycelium. *Sordariales* species usually remain sterile, only chlamydospores or large swollen hyphae being observed [4]. Culture differences can be used for approximate differentiation of agents of the three orders, while identification down to the species level requires molecular techniques [5, 10, 12].

The importance of species identification in black-grain eumycetoma infection has been indicated in several studies [10, 11, 12, 13]. The pathogens differ in susceptibility to antifungal agents, as well as in their epidemiology [12, 14]. For example, in *Pleosporales*, *Medicopsis romeroi* showed low susceptibility to several antifungal agents including amphotericin B, fluconazole, itraconazole and caspofungin [14, 15, 16]. In addition, differences are noted between the closely interrelated species of *Madurella* in their susceptibility to the main drug used for treating eumycetoma, itraconazole. While *M*. *mycetomatis*, the predominant species worldwide, is inhibited by low concentration of itraconazole, *M*. *fahalii* has high Minimum Inhibitory Concentrations (MICs) to this drug [12, 17]. Of note, the epidemiology of the two species may be overlapping, as both species are found in Sudan [12, 18].

Sudan is a hyperendemic region for mycetoma and it is considered as the disease homeland [2, 19]. The incidence of newly diagnosed cases per year in the Mycetoma Research Centre in Khartoum is 355 [18]. Of the 6,792 patients reported with myceoma in this center, 2,476 patients (37%) were from Gezira State, Central Sudan [18]. Therefore, in 2012, the Gezira Mycetoma Center was established to provide medical care for mycetoma patients in that region. During the period January 2013 to December 2016, a total of 1,654 patients were seen at the Gezira Mycetoma Center; of them, 584 underwent surgery, including amputation in 71 patients (12.2%). Diagnoses were made by clinical evaluation, imaging, fine-needle aspiration cytology and histology [20].

Although culture and molecular identification of the causative pathogen is considered the gold standard for diagnosis of mycetoma, culturing is rarely performed even in highly specialized centers [10, 5]. One of the reasons is that mycetoma agents require several weeks to grow, and risks of absence of growth or contamination are very high [10, 21]. Recently, culture-free methods have been introduced for direct detection of agents of eumycetoma in clinical materials. These include recombinase polymerase amplification (RPA), loop-mediated isothermal amplification (LAMP), and direct PCR amplification followed by sequencing [22]. Despite the high accuracy of these methods, none of them has been installed for routine diagnostics in endemic regions.

In the present study, we applied direct PCR and sequencing for diagnosis of black-grain eumycetoma cases seen at the Gezira Mycetoma Center. Since *M*. *mycetomatis* is known as the main agent of mycetoma in Sudan, we also applied species-specific PCR using primers described by Ahmed *et al*. [25]. Furthermore, we studied the epidemiology of the disease in the Gezira State based on stored information on the patient population.

## Materials and methods

### Ethical considerations

The ethical approval for this study was obtained from Faculty of Medical Laboratory Sciences, University of Gezira and from The Ministry of Health, Sudan. Since the majority of the patients cannot read or write, verbal consents were obtained before collecting the data and specimens. The consent was approved by the ethical review board. In case of children the consent was taken from parents. Data was processed and analyzed anonymously.

### Study area and population

The study was conducted at the Gezira Mycetoma Center (GMC, Sudan), which is a specialized center for mycetoma diagnosis and treatment. Patients admitted to the center were mainly from Gezira and neighboring States (Sinnar State), but patients from other parts of Sudan were also seen. Gezira State is located in the central-eastern of Sudan, about 116 km from Khartoum, and it is bordered to the South by Sinnar State. Gezira State comprises eight localities with estimated population of 4.9 million, while Sinnar State comprises seven localities with approximately 1.5 million inhabitants. All patients clinically suspected to have mycetoma and underwent surgical intervention between January 2015 and January 2017, were included in the study. Mycetoma was defined by the presence of grains in a discharge or biopsy. In the absence of visible grains, histological sections were used to confirm mycetoma. Data on demography, and clinical information of the patients were collected.

### Sample collection and processing

Biopsies were collected from the patients and the color of the grains was recorded. Of note, the surgical procedure was performed as a part of treatment and diagnostic purposes. Samples with black grains were further processed for specific identification of the etiologic agent. For 17 patients, samples were inadequate and thus were used for diagnostic purposes only. Ninety-four black-grain specimens were collected from excised mycetoma biopsies during surgery and transferred into sterile physiological saline solution. Grains were washed immediately for several times to remove tissue remnants, blood and inflammatory exudates. The grains were stored in the saline solution at -20 °C until used.

### Genomic DNA extraction

**Fig 1:**
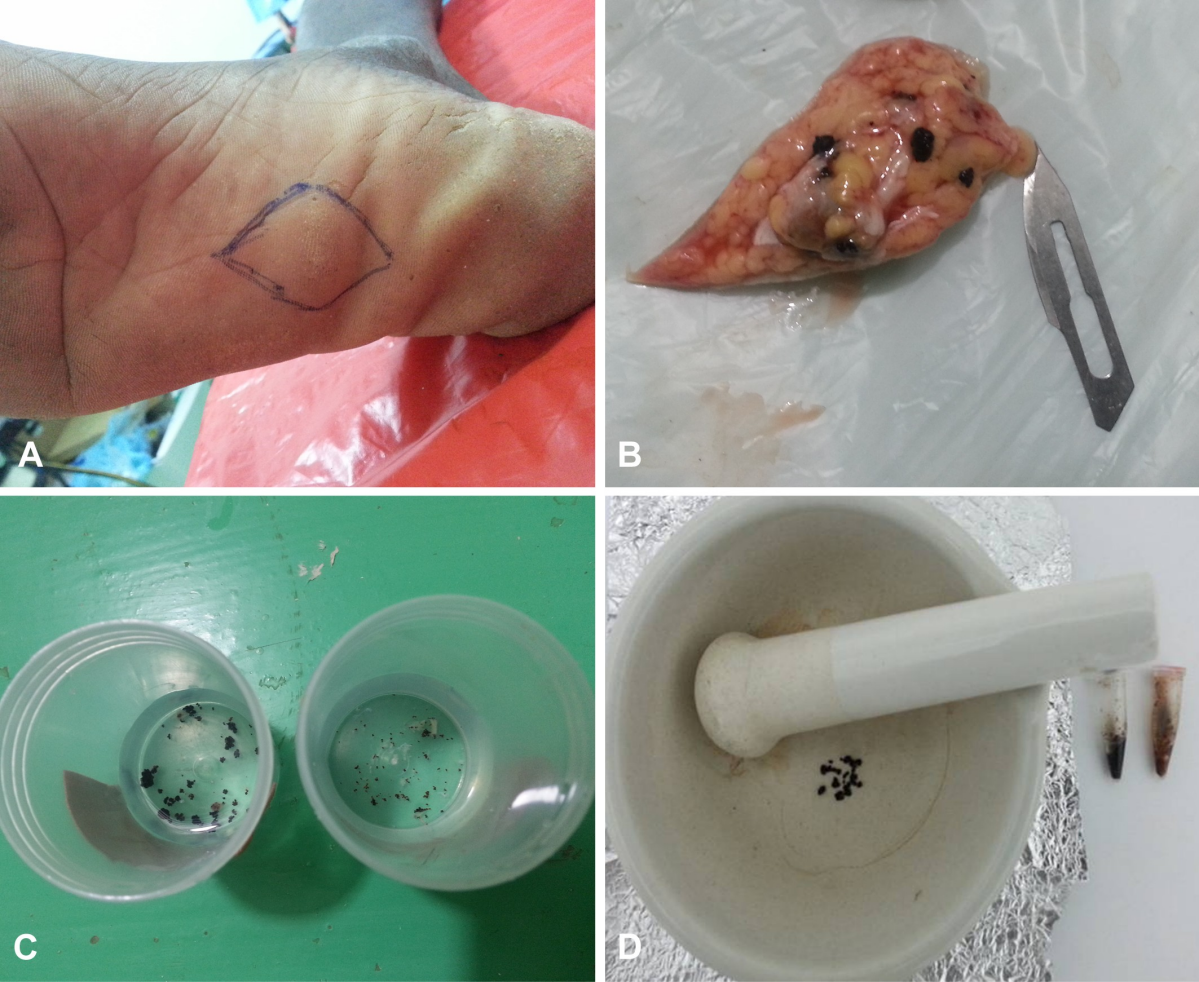
Sample collection and homogenization (A) Mycetoma lesion, (B) Deep excised biopsy, (C) Black-grains specimen, (D) Manual homogenization of the black-grains.

Approximately, 0.5 to 2 g of black-grain material was transferred to a sterile mortar, ground to fine pieces with pestle, and scrapped into a sterile 2 mL Eppendorf tube (Fig 1). CTAB (cetyl trimethyl ammonium bromide) /chloroform-isoamyl alcohol extraction protocol for fungi was followed [24]. In the procedure; 490 µL freshly prepared CTAB buffer and 10 µL of proteinase K (iNtron, Seongnam, Korea) were added to the black-grain material. Mixture was homogenized by vortexing for few minutes and then incubated for one hour at 60°C. After incubation, tubes were shaken to ensure mycelial disruption. An amount of 500 μL of chloroform: isoamyl-alcohol (CDH) (24: 1) were added to the tubes followed by shaking again for 1–2 minutes to form an emulsion. The tubes were spun at 14,000 rpm in a microfuge for 10 minutes. The upper aqueous layer was collected in a new sterile Eppendorf tube containing 0.55 volume ice-cold iso-propanol (stored at –20°C) and the tubes were spun again. Finally, the DNA pellets were washed with 70% ethanol, air-dried and re-suspended in 100 μL TE buffer (10 mM Tris, 1 mM EDTA, pH 8.0). The DNA was stored frozen at –20°C. DNA concentration (µg/mL) and purity were determined using NanoDrop spectrophotometer (Bibby Scientific, UK) at 260 nm wavelength and (OD) ratio at wavelength of 260/280. DNA quality was also assessed in agarose electrophoresis.

### PCR and sequencing

Extracted genomic DNA was amplified using universal fungal primers ITS4/ITS5 and *Madurella mycetomatis* species-specific primers, 26.1A /28.3A (Eurofins Genomics, Ebersberg, Germany) [23, 25]. The reactions were performed using iNtRON's Maxime PCR PreMix Kit with reaction volume of 20 μL that contains in addition to Maxime PCR PreMix: 3 μL genomic DNA, 0.8 μL reverse primer, 0.8 μL forward primer (10 pmol each) and 15.4 μL distilled water. PCR were performed using GeneAmp PCR system 9700 (Applied Biosystems) with the following conditions: primary denaturation (95 for 2 min), then 35 cycles of alternating denaturation (94°C for 30 s), annealing (58°C for 30 s), and extension (72°C for 30 s) with final extension for 2 min. The resulted amplicons were visualized in 1.5% agarose gel. For *M*. *mycetomatis* specific PCR, a positive control (*M*. *mycetomatis* isolate previously identified by sequencing of ITS) that showed a band size of approximately 420 bp was used. For ITS PCR, 71 randomly selected amplicons were chosen for sequencing using primers ITS4 and ITS5. Standard Sanger sequencing was performed by Macrogen (Seoul, South Korea).

### Sequences alignment and strains identification

Sequence data were assembled using BioEdit 7.2.5 [26] and the identity was accomplished using BLAST in GenBank. To confirm the identity of the causative pathogens, we assessed the phylogeny of our sequences to sequences from three reference collections, *i*. *e*. the CBS-KNAW reference collection of the Westerdijk Fungal Biodiversity Institute, The Netherlands; the Institute Pasteur Collection of Fungi (UMIP), France; and the National Reference Center for Invasive Mycoses and Antifungals (NRCMA), France. The multiple-sequence alignments were generated using MUSCLE [27]. Phylogenetic trees were constructed with MEGA v7.0.18 using maximum likelihood approach with Kimura 2-parameter model [28].

### Statistics

Descriptive analysis was performed to determine the frequency of patients sex, ages, localities and infected sites as well as the frequency of identified *Madurella* species.

## Results

### Demographic and clinical data

**Fig 2:**
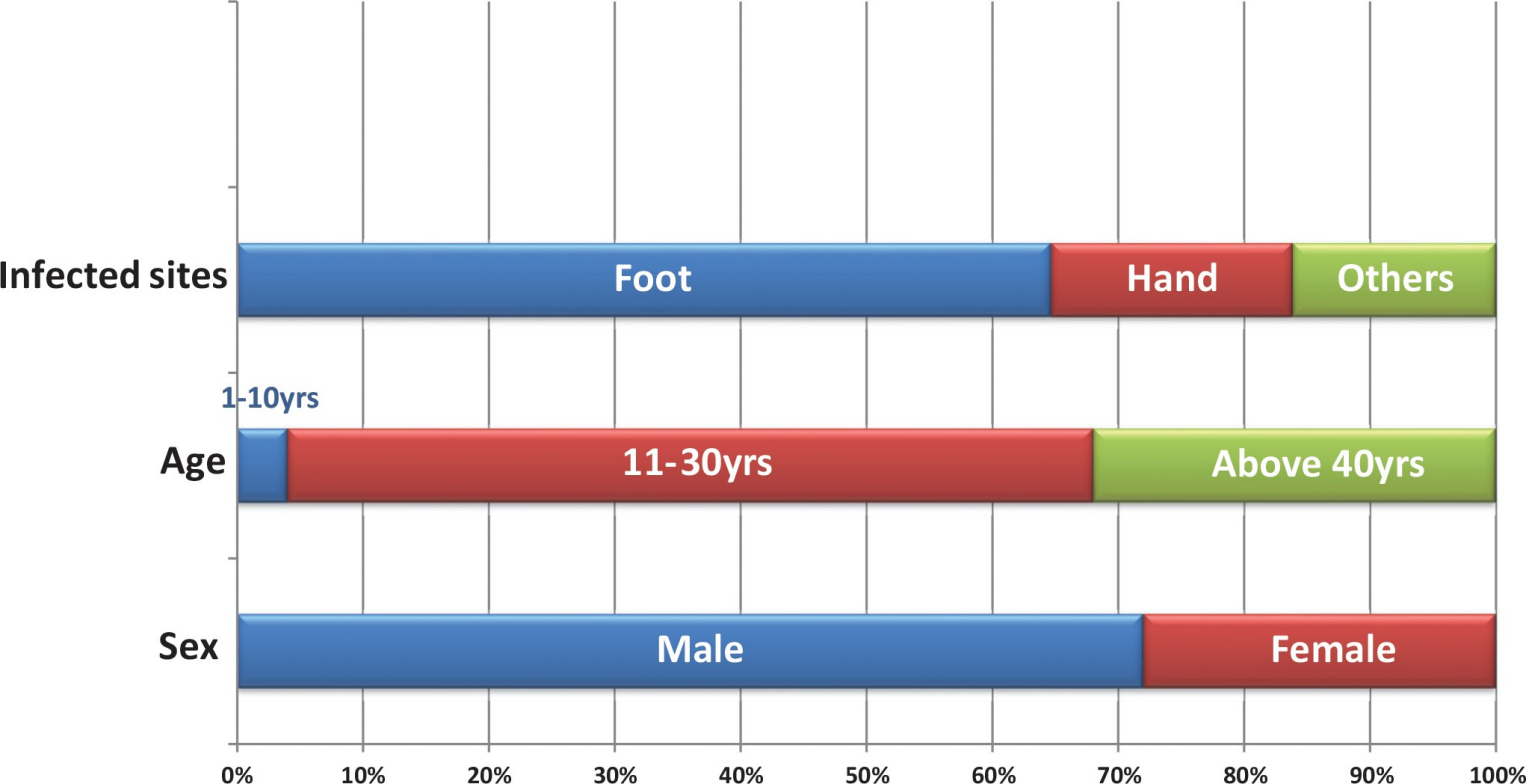
Frequency of sex, age groups and infected body sites among studied mycetoma patients .

**Fig 3:**
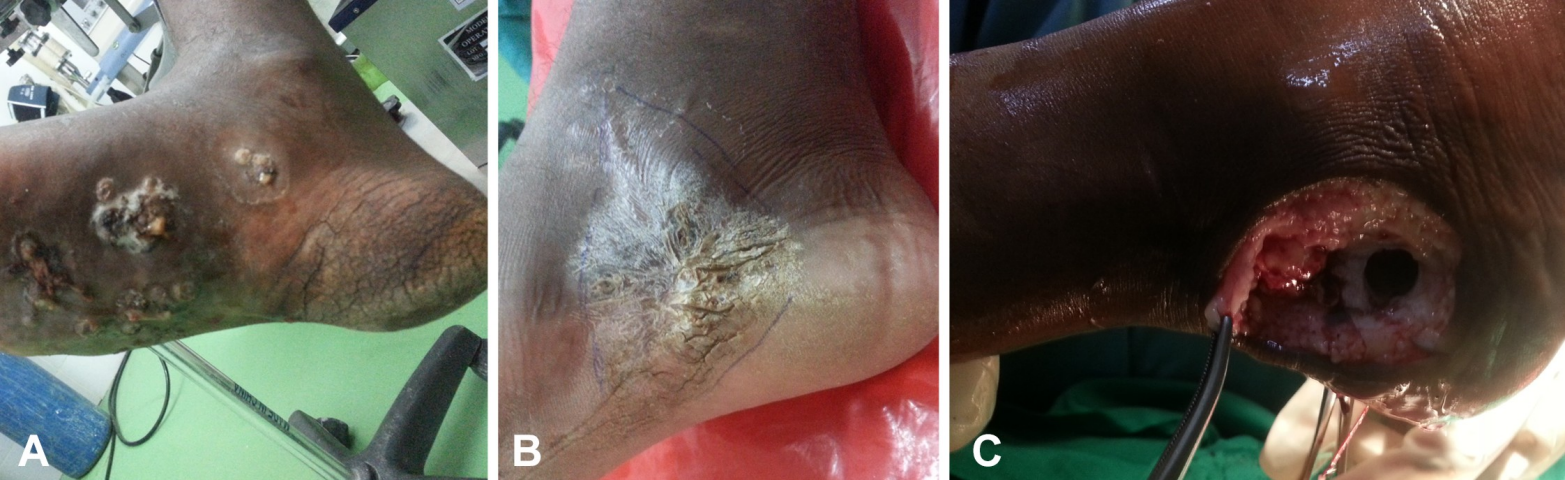
Clinical presentation of mycetoma cases . (A) Advance cases with multiple sinuses, (B) Recurrent mycetoma, (C) Bone involvement.

In total, 138 patients were suspected to have mycetoma and underwent surgical intervention during the study period. The number of confirmed mycetoma cases was 119 (86.2%); of them 86 were male and 28 female, the ratio being 2.6:1. The most affected age group was from 11 to 30 years, while the overall range was from 1 to 71 years old. Affected body sites included: foot 70% (n = 83), hand 16% (n = 19), leg 4% (n = 5), knee 3% (n = 4), back 2.5%, (n = 3), bottom 2.5% (n = 3) and perianal 2% (n = 2) (Fig 2). Patients presented with different stages of the disease ranging from early and small lesions to massive lesions and bone involvement (Fig 3). Bone involvement, mycetoma relapse and positive family history were recorded in 21% (n = 25), 12% (n = 14) and 12% (n = 14) of cases, respectively.

**Fig 4:**
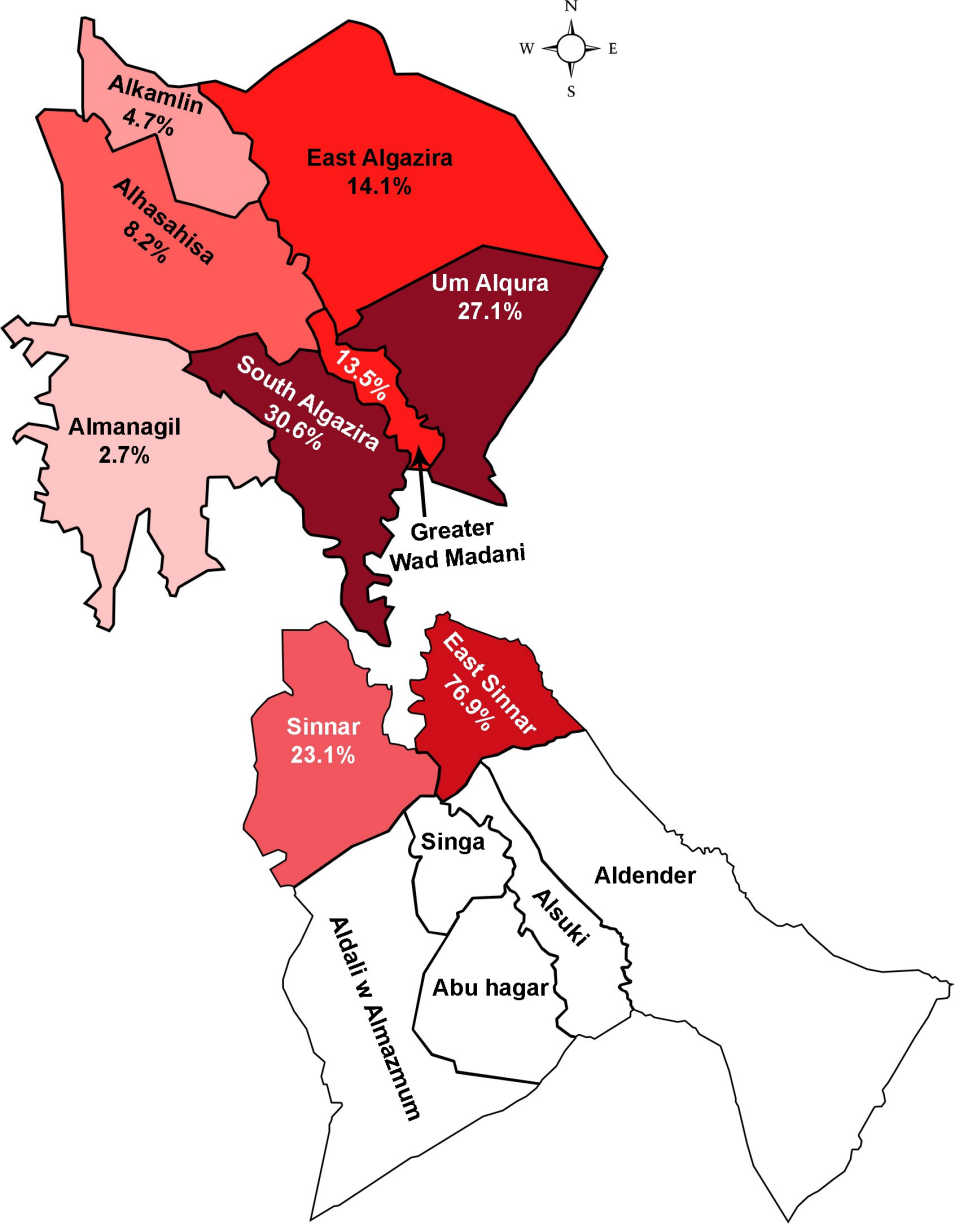
Frequency of black-grain mycetoma in Gezira and Sinnar States . **Upper left:** Gezira State localities**, Lower right:** Sinnar State localities.

Black-grain mycetoma constituted 93.3% (n = 111/119) of the cases, 76.6% (n = 85/111) were from Gezira State and distributed among the eight localities. Highest number of cases was from South El Gezira: 30.6% (n = 26/85) and from Um Elqura: 27.1% (n = 23/85), while El Managil showed the lowest rate with 2.4% (n = 2/85). Black-grain mycetoma cases from Sinnar State were 23.4% (n = 26/111) and came from two localities: East Sinnar 76.9% (n = 20/26) and Sinnar 23.1% (n = 6/26) (Fig 4).

### Molecular identification

In the present study, a total of 94 black-grain specimens were processed for direct fungal DNA extraction following the CTAB protocol. Of them, 3 samples were lost during processing while 91 samples were maintained. The purity of the extracted DNA was evaluated and 75.8% (n = 69) of the samples had 260/280 ratio between 1.6–2.0; for 15 (16.5%) samples the ratio was <1.6, while for 7 (7.7%) samples the ratio was >2. Time required for extraction including the homogenization step was approximately 1.5 hrs.

**Fig 5:**
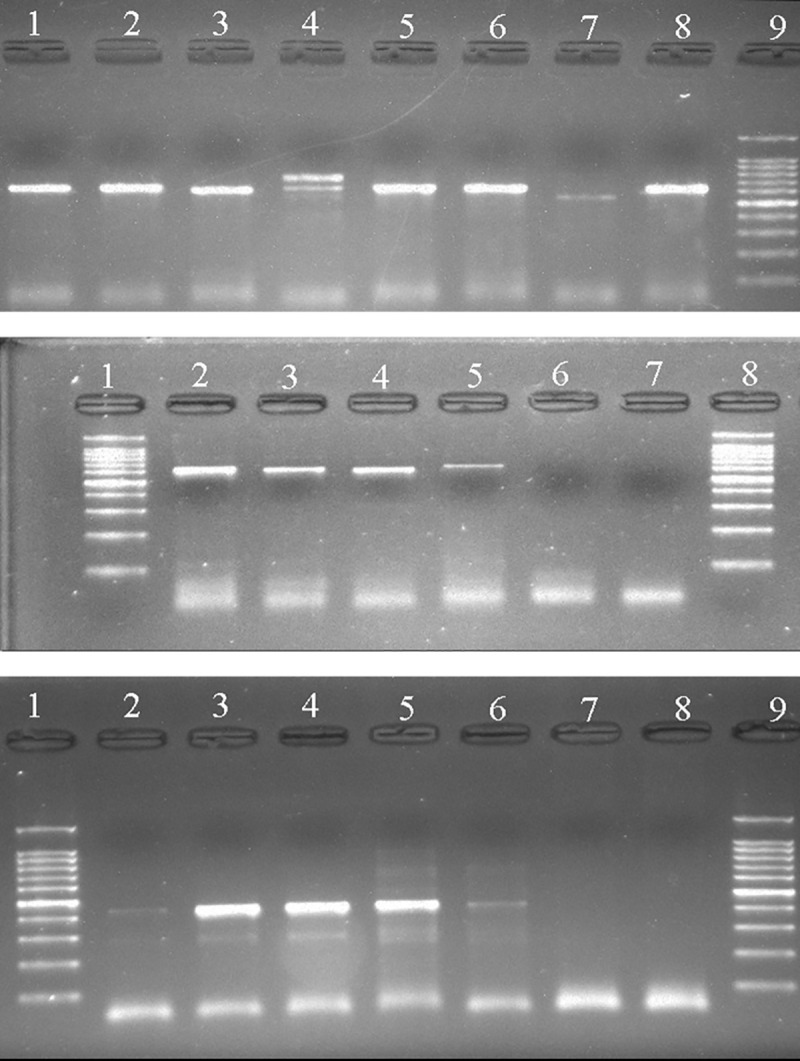
Gel representation of direct ITS and *Madurella mycetomatis*-specific PCR. **(A)** PCR of the ITS region using ITS4/ITS5 primers. A double band was noted in sample 4; **(B)** PCR of the ITS region. Lanes: 1 DNA ladder, 2 *M*. *mycetomatis*, 3 *M*. *tropicana*, 4 *M*. *fahalii*, 5 *Sphaerulina rhododendricola*, 6 and 7 are negative; **(C)** PCR using *Madurella mycetomatis*-Specific primers. Lanes: (2–6) *M*. *mycetomatis*, 7 *M*. *tropicana*, 8 *M*. *fahalii*.

Of the 91 DNA specimens, 90.1% (n = 82) were successfully amplified with the ITS primers and yielded amplicons of approximately 600 bp. Negative specimen (n = 9) couldn’t be amplified even after using other universal primer for ribosomal DNA amplification, namely V9D and LS266 [25]. Furthermore, one sample showed double DNA bands (Fig 5). By using *Madurella mycetomatis* species-specific primers, 79 specimens (86.8%) were successfully amplified and showed positive results with band sizes of approximately 420 bp (Fig 5). Only 3 specimens were positive with ITS PCR and negative with *Madurella mycetomatis* species-specific primers.

**Fig 6:**
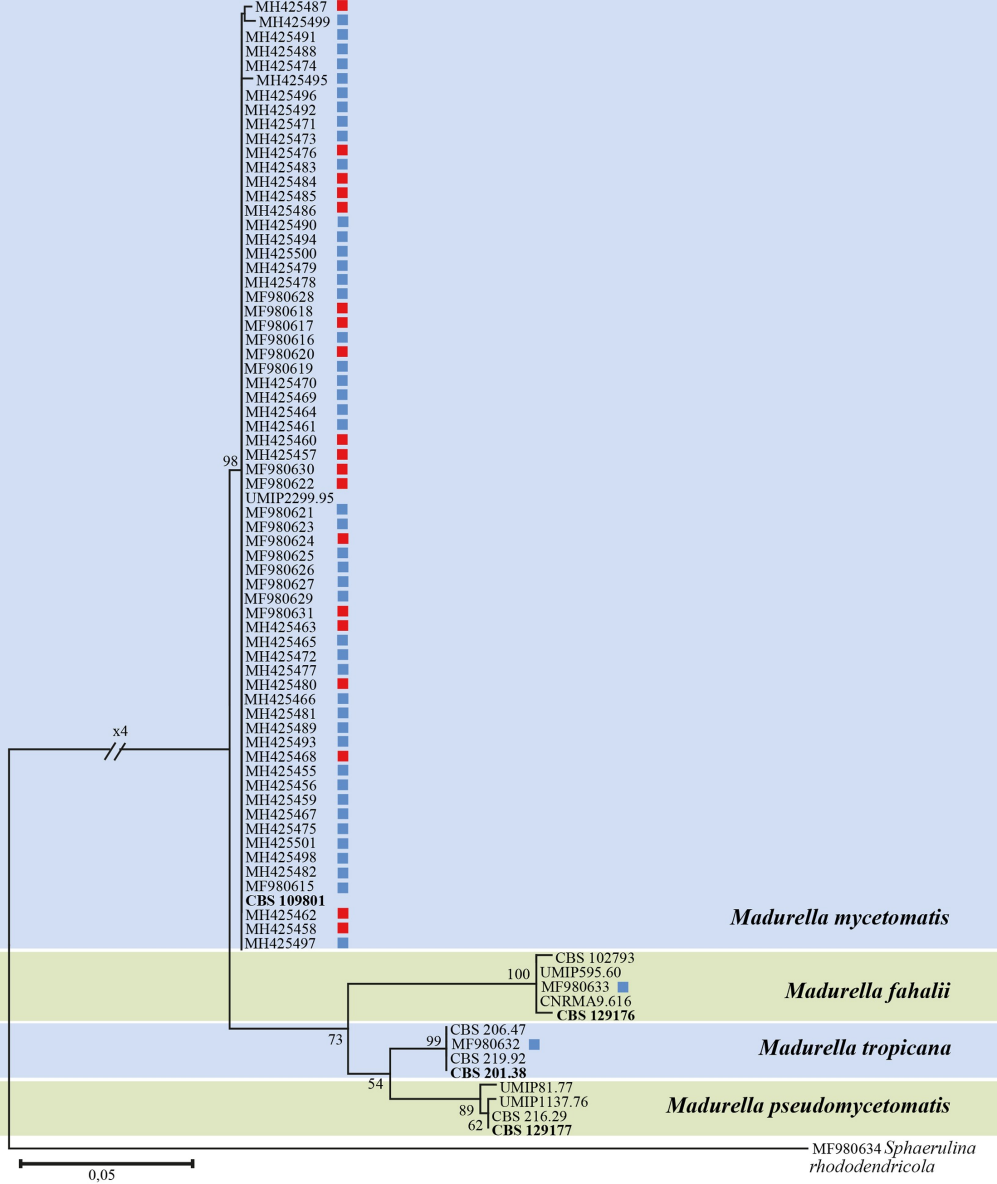
Maximum likelihood tree of ITS sequences obtained from black-grain samples. Blue square, samples from Gezira; red square, samples and from Sinnar. Type strains are shown in bold.

ITS sequencing was performed for 68 randomly selected samples, in addition to the three samples which showed positive ITS PCR but negative *M*. *mycetomatis* species-specific PCR. Sequences were deposited in GenBank database under the accession numbers shown in Fig 6. Of the 71 samples, 67 sequences were obtained and analyzed, while for 4 samples no electropherogram was obtained. With the BLAST search using ITS sequences, *M*. *mycetomatis* was identified in 64 samples which is in 100% agreement with *M*. *mycetomatis* species-specific PCR. Of the three *M*. *mycetomatis* pecies-specific PCR negative samples, one was identified as *M*. *tropicana* and one as *M*. *fahalii*. One sample didn’t match any of the known mycetoma causative species, instead, it showed 99.37% similarity to the plant pathogen *Sphaerulina rhododendricola* (ITS accession of the type strain, NR_137839).

An ITS alignment was constructed for 80 sequences with total length of 544 bp including gaps. The species of *Madurella* were clearly separated into 4 clades each representing a single species (Fig 6). The largest clade contained samples that showed identity to the type strain of *M*. *mycetomatis*. No intraspecific genetic differences were observed in the ITS of *M*. *mycetomatis* samples of the patients from different areas in Gezira State. In agreement with BLAST results, we found one sample of *M*. *fahalii* and this represents the second record of *M*. *fahalii* from Sudan. Besides the type strain of *M*. *fahalii*, other strains originated from Mali, British Columbia, and Canada. We found the first Sudanese *M*. *tropicana* case in El Managil region of Gezira State; other records of *M*. *tropicana* were from Indonesia, and Curacao. The sample that matched *Sphaerulina rhododendricola*, *Capnodiales *was at a large distance from *Madurella*, *Sordariales*.

## Discussion

This study dealt with black-grain mycetoma patients operated at the Gezira Mycetoma Center (GMC) in the years 2015 to 2017. In the study group, male patients represented 72% of the total number and the most affected age group was between 11–30 years. In Sudan, a similar observation was made by Fahal *et al*. [18] in which 76% of the mycetoma patients were found to be male and 64% were under the age of 30 years. As mycetoma is an implantation mycosis, infection of males at this age group was previously explained by an increased risk of trauma during field work, and contact with soil and thorns in agricultural settings [29]. However, since both sexes might have a similar chance of contact with the causative pathogen, another hypothesis is, that males may express a higher susceptibility to the infection [30]. Although not proven for mycetoma, the endocrinological and immunological differences between male and female was found to be determinant for susceptibility to many infectious diseases including some fungal infections [30, 31, 32]. In the present study, we found that the most frequently infected body sites were foot 70% (n = 83) and hand 16% (n = 19). In addition, we encountered two cases of uncommon, perianal infected sites; one of these patients was a horse carriage driver, while the other was a laborer. The enigmatic route of infection emphasizes the need of taking more protective measures for workers in endemic regions [7].

**Fig 7:**
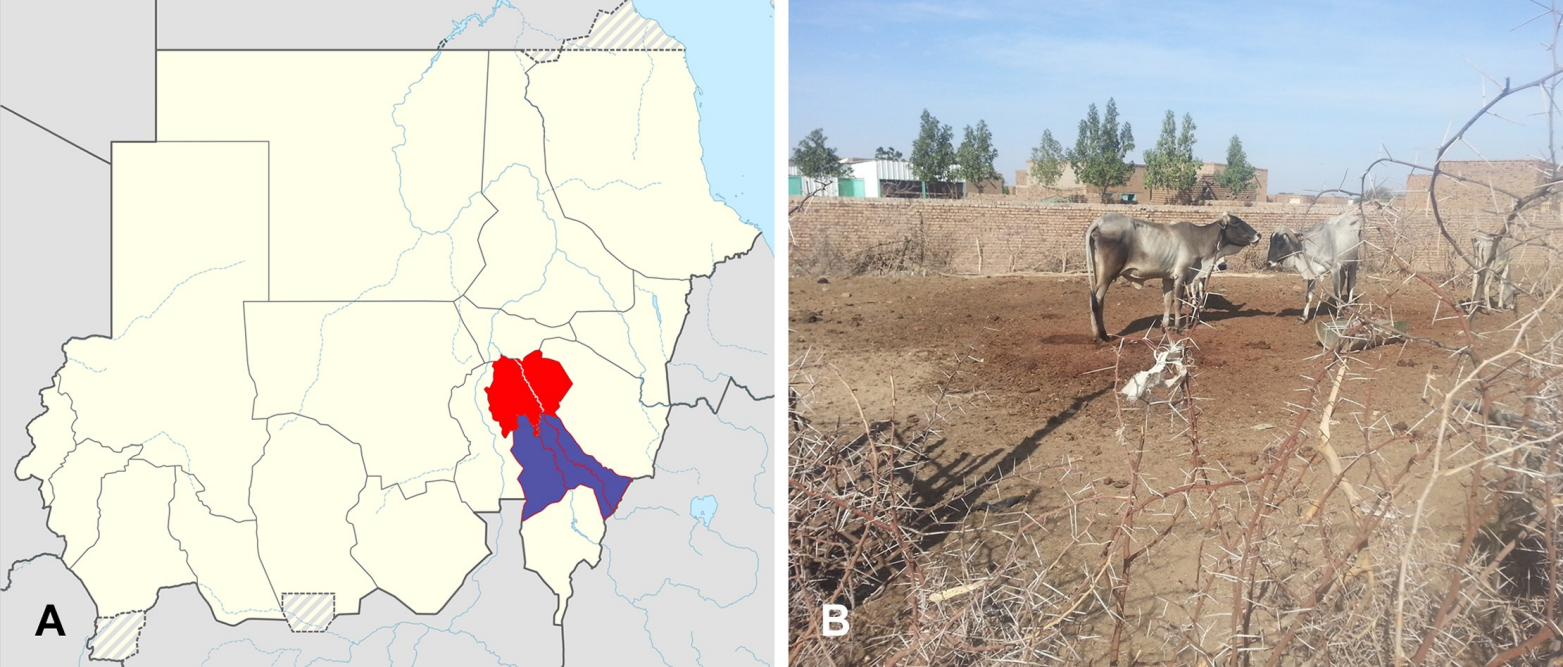
Sudan map, mycetoma endemic areas. A) Gezira State (Red), Sinnar State (Purple). B) Thorns and animal dung in an affected village in Gezira State.

Despite the large diversity of agents of black-grain eumycetoma, the present study showed that *M*. *mycetomatis* is responsible for 96% of the cases of eumycetoma in the Sudanese states Gezira and Sinnar. Its prevalence is comparable to observed frequencies of the species in the Mycetoma Reference Center in Khartoum, where *M*. *mycetomatis* was found to cause 70% of the infections; 4,754 out of 6,792 patients being infected by this fungus [18]. There is overlap in the data, as most of the cases treated in the Khartoum center (37%) were from Gezira State. In Sudan, Gezira, Sinnar, White Nile, Kordofan, and Khartoum States were the most affected regions according to data from Mycetoma Reference Center [18]. The high endemicity in these regions was explained by the ecological predilection of the causative agent in these semiarid regions [33]. Although the exact niches of *Madurella* species remains as yet unknown, ecological niche modeling revealed a correlation between *Acacia* trees and mycetoma occurrence in Sudan [33]. However, testing *Acacia* thorns from the same endemic region for the presence of *Madurella* DNA yielded negative results [34]. The role of the thorny bushes of *Acacia* might be implantation of *Madurella* into the human body, rather than being a habitat for this fungus. The phylogenetic position of *Madurella* in *Chaetomiaceae* (order *Sordariales*) has generated another hypothesis, *i*.*e*. that *Madurella* might be a coprophilous fungus, with animal dung as a possible habitat [1,7]. This hypothesis was also supported by the fact that in villages endemic for mycetoma, animals are kept in close vicinity to the houses and in sheds surrounded by thorny bushes (Fig 7) [35]. The origins of the cases reported in the present study seemed to be in line with this hypothesis, as the highest number of the cases originated from South El Gezira (23.4%) and Um Elqura (20%), which are rural regions. Greater Wad Madani is located between these provinces but only 11 cases (10%) were recovered from there. This is probably due to urbanization, as Wad Madani is the most developed, and is the economic center of the Gezira State. Of the seven locations in Sinnar, only two harbored cases of eumycetoma, with East Sinnar showing highest number of cases (18%). Interestingly, 77.5% of overall cases (n = 86/111) were aggregated in a zone of five adjacent localities of the Gezira and Sinnar States (Fig 4). These five localities have similar environmental conditions, and there is extensive traffic between the states.

Until recently, all black-grain eumycetomata caused by *Madurella* were attributed to *M*. *mycetomatis*, as this was the only accepted species in the genus beside *M*. *grisea* which now has been transferred to *Trematosphaeria* as *T*. *grisea* [6, 8, 36]. However, molecular studies have revealed larger diversity in the genus with three additional species, *viz*. *M*. *fahalii*, *M*. *pseudomycetomatis*, and *M*. *tropicana* [12, 37]. *Madurella mycetomatis* remains the prevalent species causing mycetoma worldwide, while the others are rare [2]. Of *M*. *fahalii*, a single infection has been reported from Sudan [12], but we discovered several more cases caused by this species. The one from our study concerned a patient from South El Gezira, while other cases were observed in Mali, British Columbia, and Canada (Fig 6). Furthermore, two strains of *M*. *fahalii* have been reported in patients from Saudi Arabia [38]. Given the origin of the patients infected with *M*. *fahalii*, it seems that the epidemiology of the species indeed overlap with *M*. *mycetomatis*. One of our patients was infected with *M*. *tropicana*, a species which has as yet not been reported from Sudan [12]. Thus, all *Madurella* species except *M*. *pseudomycetomatis* are present in Sudan causing eumycetoma. Given the differences in antifungal susceptibility, identification at the species level is recommended, which can be achieved by molecular methods [12]. By applying such methods, we have identified a new agent of black grain eumycetoma in Sudan, *Sphaerulina rhododendricola*. Although agents of eumycetoma are distributed into at least eight orders in the Ascomycota, there are no reports for species in the order *Capnodiales*. *Sphaerulina rhododendricola* is the first Capnodialean fungus to be reported [39]. Furthermore, there might be other species from this order, but the use of methods with insufficient specificity for diagnosis has limited the detection of such species.

Our study applies molecular, culture-free methods for the diagnosis of black-grain eumycetoma using a large sample size from hyperendemic regions in Sudan. K *et al*. [40], used direct PCR and sequencing for diagnosis of a culture-negative black-grain mycetoma case in India. One of the challenges of direct molecular detection is DNA extraction, as the grains may be recalcitrant to homogenization. Ahmed *et al*. used metal beads in a tissue lyser and a DNA extraction kit [22]. For less equipped laboratories in developing countries, we recommend cheapest extraction method with CTAB and homogenization by grinding [24]. To achieve a high DNA concentration of sufficient quality, grains should carefully be washed to remove dead tissue and inflammatory exudates. With ITS4 and ITS5, 90.1% (n = 82/91) of our samples were successfully amplified. Absence of amplification might be due to loss of DNA during purification, or to presence of PCR inhibitors. Although double bands were observed with ITS PCR in one of the samples, this sample proved to be positive with *M*. *mycetomatis* specific PCR. Of note, all our samples were from excised mycetoma biopsies during surgery, rather than from discharge through fistels, minimizing contamination. Nevertheless, evaluation of the same detection method using discharged grains is needed to verify whether the approach can be simplified abandoning invasive procedures.

In summary, we confirmed that the conclusive diagnosis of eumycetoma can be achieved by direct detection of the causative pathogen from the grains using PCR and sequencing. The method can be applied for routine diagnosis in reference centers such as the Gezira Mycetoma Center. The identification of uncommon agents of eumycetoma in this study, such as *M*. *fahali*, *M*. *tropicana*, and *S*. *rhododendricola* highlighted the importance of species identification in clinical settings. These species might differ in their susceptibility to antifungal agents and their epidemiology thus specific identification is required for both patient’s management and to properly reveal the epidemiology of the species [12].
